# Phenylpropanoids metabolism: recent insight into stress tolerance and plant development cues

**DOI:** 10.3389/fpls.2025.1571825

**Published:** 2025-06-26

**Authors:** Vincent Ninkuu, Oluwaseun Olayemi Aluko, Jianpei Yan, Hongmei Zeng, Guodao Liu, Jun Zhao, Huihui Li, Songbi Chen, Felix Dapare Dakora

**Affiliations:** ^1^ National Nanfan Research Institute, Chinese Academy of Agricultural Sciences (CAAS), Sanya, China; ^2^ State Key Laboratory for Biology of Plant Diseases and Insect Pests, Institute of Plant Protection, Chinese Academy of Agricultural Sciences (CAAS), Beijing, China; ^3^ Crops Genetic Resources Institute, Chinese Academy of Tropical Agricultural Sciences/Key Laboratory of Ministry of Agriculture for Germplasm Resources, Conservation, and Utilization of Cassava, Danzhou, China; ^4^ Biotechnology Research Institute, Chinese Academy of Agricultural Sciences (CAAS), Beijing, China; ^5^ State Key Laboratory of Crop Gene Resources and Breeding, Institute of Crop Sciences, CAAS, Beijing, China; ^6^ Chemistry Department, Tshwane University of Technology, Pretoria, South Africa

**Keywords:** phenylpropanoids, plant interactions, post-transcription, post-translation, epigenetics modifications, plant development

## Abstract

The phenylpropanoid pathway remains a key target for most climate-resilient crop development, owing to it being a precursor to over 8000 metabolites, including flavonoids and lignin compounds, including their derivatives. These metabolites are involved in biotic and abiotic stress tolerance, inviting several studies into their roles in plant defense, drought, temperature, UV, and nutrient stress tolerance. Literature is currently inundated with cutting-edge reports on the phenylpropanoid pathways and their functions. Here, we provide a comprehensive update on the biosynthesis of phenylpropanoids, mainly lignin and flavonoids, their roles in biotic and abiotic interaction, and transcending topics, including pest and diseases, drought, temperature, and UV stress tolerance. We further reviewed the post-transcriptional, post-translational, and epigenetic modifications regulating phenylpropanoid metabolism and highlighted their applications and optimization strategies for large-scale production. This review provides an all-inclusive update on recent reports on the metabolism of phenylpropanoids in plants.

## 1 Introduction

Phenylpropanoids are highly diverse secondary metabolites derived from the shikimate pathway, emanating from the glycolysis and the pentose phosphate pathways routes (Lehari and Kumar, 2024). The phenylpropanoid pathway branches into two, producing numerous lignin- and flavonoid-related metabolites, which are ubiquitous in the plant kingdom and greatly contribute to plant environmental interactions. Phenylpropanoids and other phenolic compounds formation commences with L-phenylalanine, an aromatic amino acid, and L-tyrosine in some grasses. An enormous array of plant self-serving metabolites are generated via the phenylpropanoid metabolic pathway through a few shikimate pathway intermediates (Siebeneichler et al., 2024). The resultant hydroxycinnamic acids and esters are converted by a series of oxygenases, reductases, and transferases, yielding developmental- and environmental cues-specific metabolites (Ninkuu et al., 2023c). Glycosides of phenylpropanoid exhibit a variety of bioactivity, including antioxidant effect, immunomodulatory effects, and enzyme-inhibitory effect (Pinar and Rodríguez-Couto, 2025).

Phenylpropanoids are categorized into several classes, including simple phenylpropanoids such as cinnamic and *p*-coumaric acids, ferulic, caffeic, and sinapic acids; phenolic acids (hydrocinnamic and hydroxybenzoic acids); flavonoids (flavones, flavonols, flavanones, anthocyanins, isoflavonoids, etc.); lignin and lignans; coumarins, and stilbenoids (Dixon et al., 2002).

Recent studies have comprehensively elucidated the molecular regulation of phenylpropanoids, diversity, and plasticity. Additionally, the role of phenylpropanoid metabolites in biotic (plant diseases and pest control) and abiotic stress (drought, temperature, UV, nutrients, etc.) are interactions continuously changing the face of climate-resilient germplasm development in recent times. Moreover, phenylpropanoids such as lignin are required for mechanical support for plant growth and the promotion of water and mineral uptake and partitioning in plants (Uddin et al., 2024). The current article provides a comprehensive update on the biosynthesis of phenylpropanoids, mainly lignin and flavonoids, their roles in biotic and abiotic interaction, and topics, including pests and diseases tolerance, drought, temperature, nutrient signaling and uptake, and UV stress tolerance. We also examined post-transcriptional, post-translational, and epigenetic modifications involved in phenylpropanoid biosynthesis and highlighted their industrial applications as well as optimization strategies for large-scale production. This review provides an all-inclusive update on recent reports on the metabolism of phenylpropanoids in plants.

## 2 Overview of the phenylpropanoid pathway

**Figure 1:**
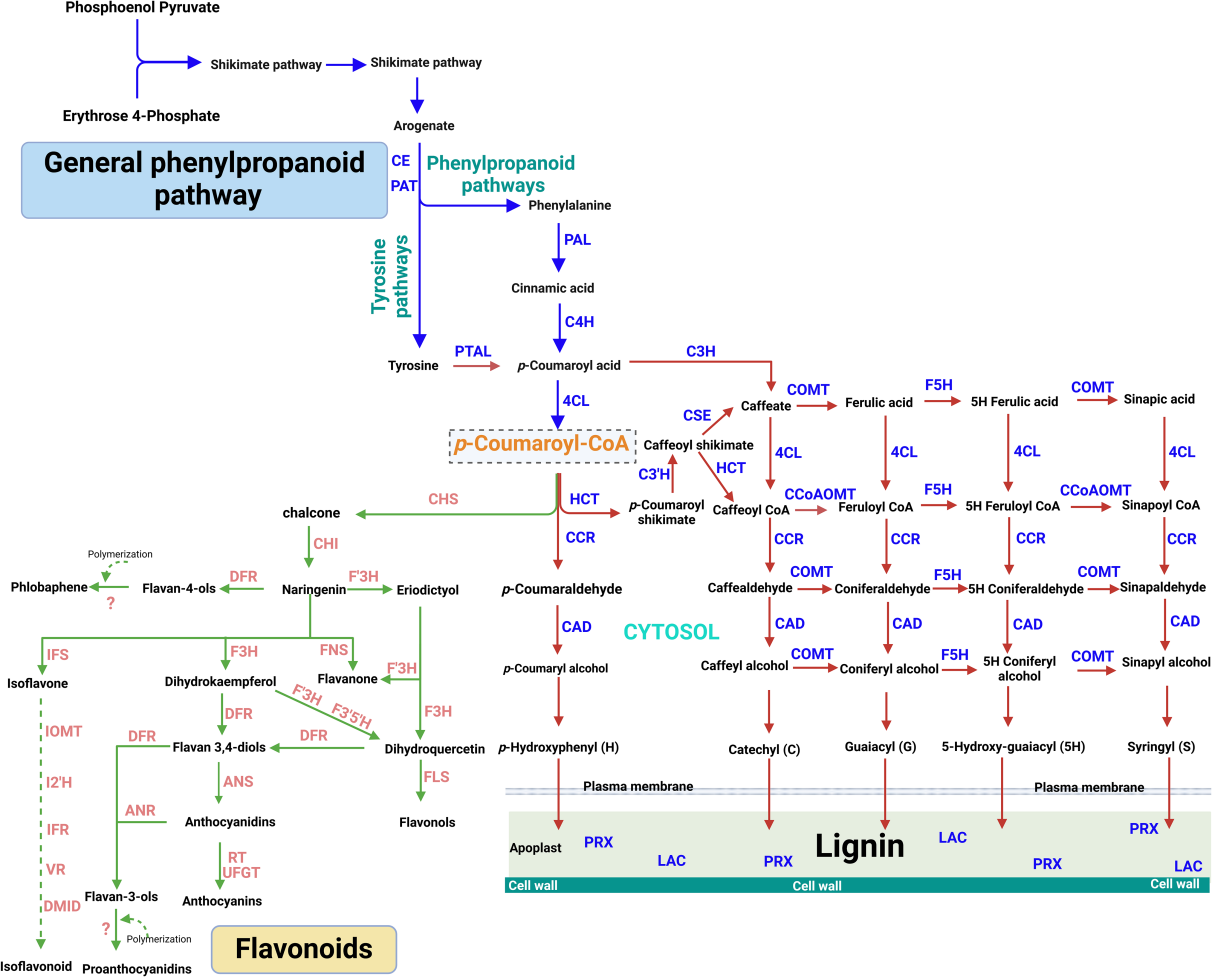
Phenylpropanoid metabolic pathway.

The intracellular, plastidial localization, and the intricate regulation of the phenylpropanoid pathway have been explored for decades now, with almost all the pathway genes and proteins identified. Whereas tryptophan, phenylalanine, and tyrosine are useful aromatic amino acids synthesizing proteins, they are also precursors to several natural products, including hormones, pigments, alkaloids, and cell wall components. Intriguingly, all three are derivatives of the shikimate pathway, where approximately ≥30% of photosynthetic carbon is fixed on plants, providing essential diet components to humans and animals due to the loss of this pathway in their lineage (Maeda and Dudareva, 2012). The shikimate, which is a crucial precursor to the phenylpropanoids pathways, is driven by a seven-step pathway characterized by six enzymes and initiated via the condensation reaction of phosphoenolpyruvate and erythrose-4-phosphate. Notably, the phosphoenolpyruvate and erythrose-4-phosphate are also derivatives of glycolysis and the pentose phosphate pathways, respectively (Ren et al., 2024; Tzin and Galili, 2010). The formation of Arogenate from shikimate is the major biosynthetic route of phenylalanine and tyrosine, encoded by prephenate aminotransferase (*PAT* and *CE*) and arogenate dehydratase (ADT). However, recent advances have also linked phenylalanine biosynthesis to phenylpyruvate in microbes (Ren et al., 2024; Tzin and Galili, 2010) (**Figure 1**). Phenylalanine ammonia-lyase (PAL) is the gate opener for several glycosylation, acylation, hydroxylation, and methylation reactions, forming over 8000 metabolites in the phenylpropanoid pathway (Ninkuu et al., 2023a).

The phenylalanine and the tyrosine in some grasses diverge into different pathways, from Arogenate but reconverges, yielding *p*-coumarate, which is a precursor to coumaroyl CoA for the formation of an array of phenylpropanoid metabolites. Coumaroyl CoA is also the precursor for the lignin and flavonoid biosynthesis (**Figure 1**). Lignin is a heterogeneous phenolic polymer and the second most abundant polymer after cellulose, forming 30% of the earth’s organic carbons in the biosphere. The so-called heterogeneity of lignin results from its polymerization from various hydroxycinnamoyl alcohol derivatives. It is subsequently deposited in the cell walls of vascular plants, conferring many stress tolerance traits, including resistance to diseases and pests, drought, deterioration, heat stress, UV radiation, etc (de Oliveira et al., 2025; Ninkuu et al., 2022). Elsewhere, we comprehensively reviewed the 11 enzymes involved in lignin biosynthesis, the phytoalexins they produced, and their individual or collaborative roles in plant immunity induction (Ninkuu et al., 2023a).

**Figure 2:**
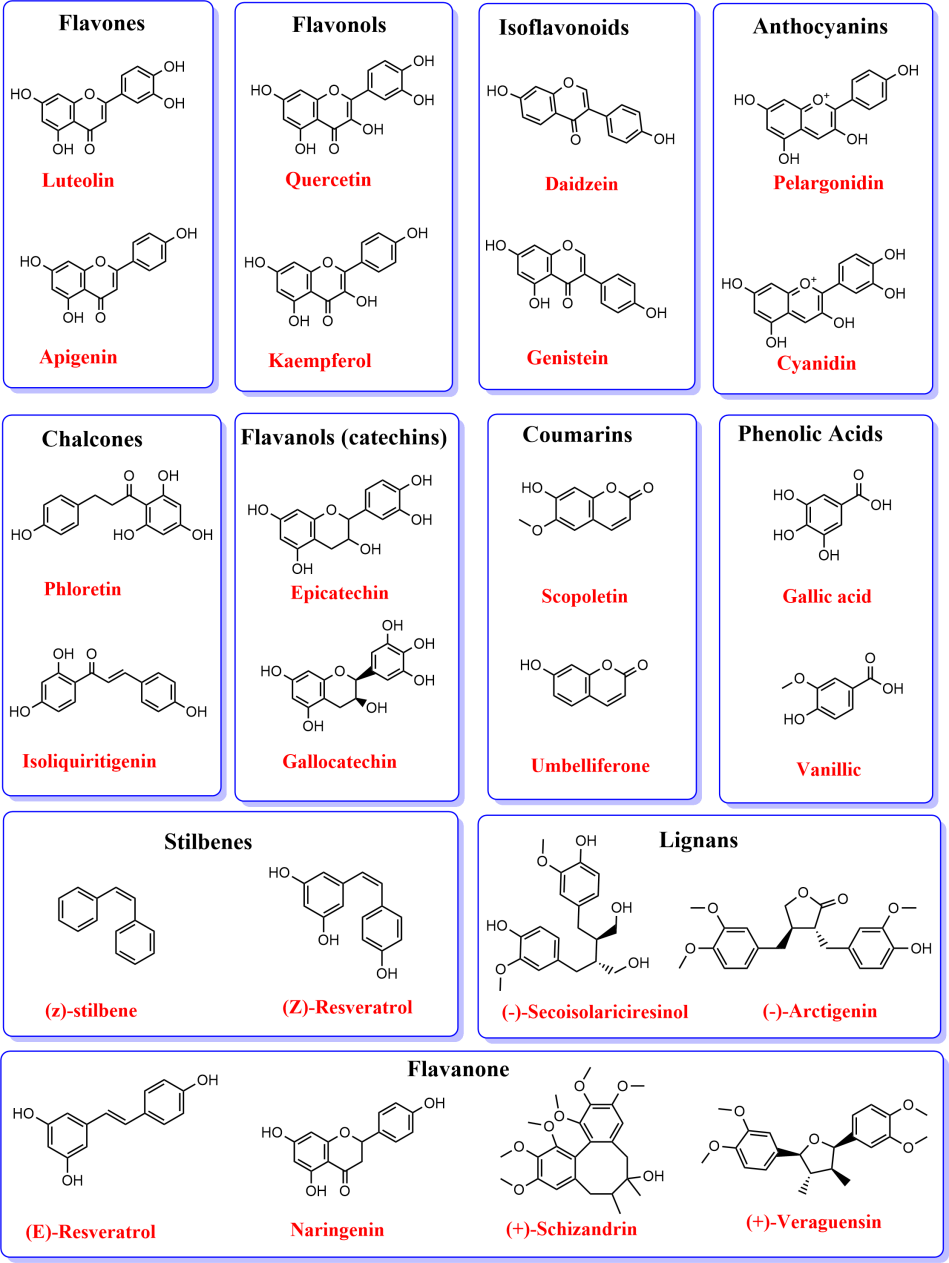
Structural formulae of phenylpropanoid metabolites.

Like lignin, flavonoid metabolism is the second branch of the phenylpropanoid pathways, producing over 6000 polyphenolic metabolites (Jie et al., 2023). Flavonoids are bioactive metabolites involved in plants’ biotic and abiotic interactions, including microbial signaling, allelopathy, and nutraceuticals for improved health (Oro et al., 2025; Zheng et al., 2025). Flavonoids are characterized by C6-C3-C6 diphenylpropane skeleton, where three carbon chains (C3) links the two aromatic rings (Shanker and Rana, 2025). Flavonoids are classified based on the heterocyclic C‐ring, such as chalcones, aurones, flavones, isoflavones, flavanones, dihydroflavonols, anthocyanidins, leucoanthocyanidins, flavonols, and flavan‐3‐ols (Chen et al., 2023). **Table 1** and **Figure 2** show the classifications of flavonoids and their structural forms, respectively. The first committed step in flavonoid biosynthesis is catalyzed by chalcone synthase (CHS), converting *p*‐coumaroyl‐CoA to chalcone, which directs the metabolic flux to flavonoid metabolism. Stilbene synthases (STS) also encode the formation of simple stilbenes from cinnamoly‐CoA and *p*‐coumaroyl‐CoA. Liu et al. (2021) review discusses the biosynthesis processes of flavonoids in plants, dissecting the various enzymes involved.

**Table 1:** Classification of phenylpropanoids.

Class	Examples	Functions
Simple Phenylpropanoids	Eugenol, Chavicol	Antimicrobial, antioxidant
Phenolic Acids	Gallic acid, caffeic acid	Defense, antioxidant
Flavonoids	Quercetin, anthocyanins	UV protection, pollinator attraction
Lignins/Lignans	Pinoresinol, lignin polymers	Structural support, pathogen defense
Coumarins	Scopoletin, umbelliferone	Antifungal, allelopathy
Stilbenes	Resveratrol	Antioxidant, antifungal
Tannins	Proanthocyanidins, tannic acid	Herbivore defense, soil nutrient cycling
Chalcones	Phloretin, isoliquiritigenin	Allelopathy, pathogen defense

**Table 2:** Role of phenylpropanoids in pests and disease mitigation.

Plants	Disease	Genes/Proteins	Metabolite accumulation	Defense activation	References
Sugarcane	Sugarcane white leaf (SCWL)	*CAD*, *CCR*, *REF1*, *POD*, *PAL*, and *HCT*	Flavonoids, lignin, and coumarins	*Candidatus Phytoplasma* sacchari	(Lohmaneeratana et al., 2024)
Sunflower	Sunflower wilting	*PAL*, *4CL2*, *CCR*, *POD10*, and *POD11*	Anthocyanins, coumarins, lignans, flavonoids, phenols	*Orobanche cumana*	(Huang et al., 2022)
Alfalfa	Curling, yellowing, and atrophy	*PAL*, *4CL* and *F6H*	Flavonoids, lignin, coumarins and phenols	Response to aphid infestation.	(Liu H. et al., 2024)
*Bambusa*	Shoot blight	*CCoAOMT2* and *CAD5*	Reduced flavonoids and lignin	Shoot blight defense	(Luo et al., 2022)
*Zanthoxylum armatum*	Pepper rust	*CHS*, *CHI*, and *DFR*	Flavonoids	Resistance against *Coleosporium zanthoxyli*	(Han et al., 2023)
–	Dodder inhibition	*PAL*, *CCR*, and *CCoAOMT*	Flavonoids, phenols, and lignin	Defense against Cuscuta japonica	(Guo et al., 2022)
Cotton	fungal infection	*PAL*, *F6’H*, and *CCoAOMT*	Phenols and lignin	Defense against *Verticillium dahliae*	(Zhang M. et al., 2024)
Korla	Blackhead disease	*PAL*, *C4H*, and *4CL*	Phenols	Resistance to *Alternaria alternata*	(Sun et al., 2025)
Wild mungbean	Root-knot nematodes	*PAL* and *POD*	Phenols	Improved resistance to *Meloidogyne* spp	(Lee et al., 2024)
*Solanum habrochaites*	Not stated	*SlCHI*, *SlHCT*, and *SlCAD*	Reduced Phenolics	Suppression of mites	(Wang et al., 2024a, b; Wang M. et al., 2024)
*Lycium barbarum* L.	Root rot	*PAL*, *4CL* and *C4H*	Lignin and flavonoids	Enhance *Rhizophagus intraradices* defense	(Li N. et al., 2024)
Cucumber	Fusarium wilt	*CHS*	Phenolics and flavonoid	Promotes plant resistance to *Fusarium wilt*	(Yang et al., 2024)
Lily	Not stated	*CHS* and *PAL*	Coumaric acid and phenolics	Increased lily resistance to Aphid infestation	(Zhou L. et al., 2024)
Maize	Stalk rot	*PAL* and *C4H*	Coumaric acid and phenolics	Resistance to *F. proliferatum*	(Sun et al., 2024)
Chinese Cabbage	Not stated	*PAL* and *4CL*	Flavonoids and phenolics	Enhanced resistance to *P. brassicae*	(Wei et al., 2024)

## 3 Biological functions of phenylpropanoid-derived metabolites

**Figure 3:**
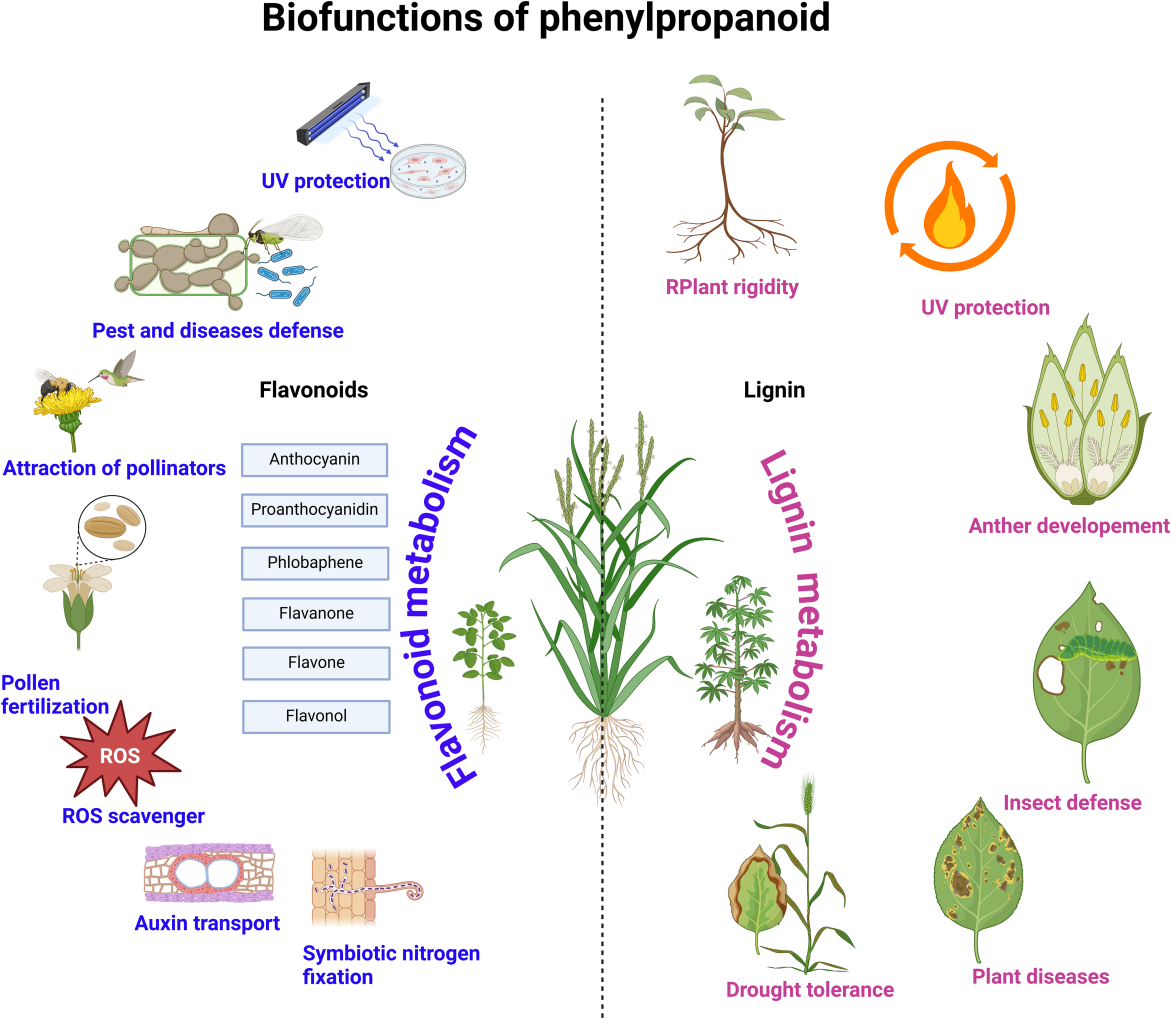
Biological functions of flavonoids and lignin.

As a sessile land organism, plants are exposed to numerous but expected environmental hazards, including pathogens and insect infections, UV radiation, drought, heat, and cold stressors. The deterioration of crop products is also quite hastened by environmental influences. Notwithstanding these unavoidable stressors imposed partly due to climate change, studies have shown that phenylpropanoid metabolism can ameliorate these factors in plants (**Figure 3**). In the following sections, we highlight recent works elucidating the role of phenylpropanoid metabolism in resisting these stresses.

### 3.1 Phenylpropanoid metabolism enhances resistance to reactive oxygen species for stress tolerance

Reactive oxygen species (ROS), including superoxides (O_2_^-^), hydrogen peroxide (H_2_O_2_), hydroxyl radical (OH^-^), and singlet oxygen species (^1^O_2_) are by-products of cellular metabolism responsive to adverse environmental stressors in plants (Rabeh et al., 2025). ROS induction signals plant growth, differentiation, and immune responses. Moreover, ROS production under stressful conditions obstructs cellular functions, leading to oxidative damage and conferring biotic and abiotic stress responses in plants. However, plants adapt to excessive ROS induction using intricate ROS-scavenging mechanisms to offset damage to protein, lipids, and DNA (Rabeh et al., 2025; Wang et al., 2024; Gao et al., 2023; Yang et al., 2023). Moreover, plants have developed sophisticated mechanisms to cope with stressors, such as phenolic compound metabolism, to neutralize ROS DNA (Rabeh et al., 2025; Wang et al., 2024).

Meeting the growing food demand presents a significant challenge to global food security, as much of the world’s arable land remains vulnerable to abiotic stresses such as salinity, drought, extreme temperatures, UV radiation, and heavy metal toxicity. Phenylpropanoid biosynthesis becomes a crucial physiological need of abiotic-stressed plants. The surge in phenylpropanoid metabolism under abiotic stress detoxifies ROS and protects cellular components from oxidative damage. Hence, crucial genes encoding key phenolic enzymes, including PAL (phenylalanine ammonia-lyase), C4H (cinnamate 4-hydroxylase), 4CL (4-coumarate: CoA ligase), CHI (chalcone isomerase), and F3H (flavanone 3-hydroxylase) are predominantly upregulated in response to various abiotic stressors (Rabeh et al., 2025; Rao and Zheng, 2025; Sharma et al., 2019).

### 3.2 Phenylpropanoids enhance plants’ tolerance to UV-B radiation

Plants exposed to UV-B stress generate harmful ROS that severely damage their DNA and proteins (Naikoo et al., 2019; Singh et al., 2023). Nevertheless, such stresses can be mitigated by increasing cellular phenolic deposition, which shields the epidermal layers of the leaves (Olson and Ruhland, 2024; Xiao et al., 2023). Phenylpropanoids further plummet DNA damage by minimizing the photodamage of crucial enzymes such as NAD/NADP, while arresting thymine dimerization (Naikoo et al., 2019). Among these phenolics, flavonoids are considered effective UV-B screening filters deposited in leaf interiors and trichomes, for plant defense against harmful radiations (Choudhary et al., 2021; Singh et al., 2023). Several studies have affirmed that a spike in flavonoid biosynthesis promotes plant tolerance to UV- radiation (**Table 3**) (Hao et al., 2022; Rizi et al., 2021; Song et al., 2025). Hence, increased expression of flavonoid biosynthetic genes (F3H, CHS, CHI, and FLS) safeguards plants against UV-B stress. According to Zhao et al. (2020), revealed that the upregulation of FLS and FS’H in response to UV-B radiation promoted flavonoid biosynthesis in Ginkgo biloba leaves. Similar upregulation of the flavonoid-induced gene (*F3H*) has been reported in a desert plant, *Reaumuria soongorica*, indicating flavonoid regulates UV-B stress adaptation (Liu et al., 2013). Martínez-Silvestre et al. (2022) revealed a higher flavonoid content in the callus irradiated with UV-B, averting the harmful effects of UV-B radiation in *Sideroxylon capiri*. Thus, flavonoids function as a “signal trigger,” neutralizing the prospective effects of UV-B light.

**Table 3:** Phenylpropanoid metabolism mediates abiotic stress tolerance in plants.

Abiotic stress	Plant species	Response to abiotic stressors	Reference
Drought stress	*Salvia miltiorrhiza*	Upregulation of *PAL*, *CAD*, *CHS*, and *4CL* enhances ferulic acid content for drought tolerance.	(Zhou Y. et al., 2024)
	*Casuarina equisetifolia*	Flavonoids and phenols accumulation improves drought stress tolerance	(Zhang S. et al., 2023b)
	*Brassica juncea* L.	*POD*, *CCoAOMT*, *4CL*, and *PAL* downregulation mediates seed germination.	(Wei et al., 2023)
	*Ligularia fischeri*	Increased expression of *CHS*, *CHI*, *F3H*, *FNS*, and *FLS* may contribute to drought tolerance.	(Park et al., 2023)
	*P. vulgaris*	A spike in isoflavone in response to drought resulted in a 50% loss of root water content.	(Peña Barrena et al., 2024)
	*Cuminum cyminum* L	Increased activity of the PAL gene indicates its significance in drought responses.	(Ghasemi et al., 2023)
	*Ophiopogon japonicus*	Changes in *4CL*, *HCT*, and *PAL* gene expression boost drought tolerance	(Cheng et al., 2025)
	*Sophora alopecuroides*	Increased flavonoid content improves root tolerance to drought	(Huang et al., 2023)
	*Adonis amurensis*	Drought stress heightens the expression of phenolics and flavonoids.	(Gao et al., 2020)
	*S*. *baicalensis*	Drought alters the expression of flavonoids in *S*. *baicalensis*	(Zhang T. et al., 2025)
	*Salvia miltiorrhiza* Bunge	Lignin deposition in the secondary cell wall safeguards plants against drought attacks.	(Zhang Y. et al., 2025)
	*Lilium brownii*	Anthocyanin accumulation improves leaf resistance to drought.	(Chen W. et al., 2025)
Salt stress	*Taraxacum officinale*	Downregulation of *ToC4H*, *To4CL*, *ToHCT*, and *ToHQT* contributes to salt tolerance.	(Zhu et al., 2022)
	*Morus atropurpurea*	Upregulation of *FLS*, *CHS*, *PAL*, and *ANR* suggests their involvement in salinity tolerance.	(Wang et al., 2024b)
	*Hordeum vulgare* L.	Lignin, flavonoids, and polyphenols in seed cells improved salinity tolerance	(Xue et al., 2023)
	*L. ruthenicum*	An increase in flavonoid content enhances salinity tolerance.	(Qin et al., 2022)
	*Chrysanthemum × grandiflora*	Upregulation of *PAL*, *CYP73A*, and *4CL* in leaves and roots improves salt tolerance.	(Liu H. et al., 2022)
	*Carex rigescens*	HCT and F5H metabolite may contribute to salt tolerance	(Wu et al., 2024)
	*Solanum lycopersicum*	Alterations of *PAL*, *C4H*, and *4CL* genes protect during salinity stress.	(Jia et al., 2022)
	*Phaseolus vulgaris*	Changes in *POD*, *4CL*, and *CCoAOMT* activities contribute to salinity tolerance.	(Zhang Q. et al., 2023)
	Phaseolus vulgaris	Salt stress enhances rutin accumulation in germinating beans	(Zhang et al., 2022)
	*Salicornia europaea*	Phenylpropanoids increase osmotic tolerance in response to salt stress.	(Duan et al., 2023)
	*Triticum aestivum* L.	Increased activities of *PAL* and *POD* protect wheat against salinity stress.	(Maslennikova et al., 2023)
	*Platycodongrandiflorus*	Upregulation of *PAL, COMT*, and *C4H* may suggest their participation in response to salt stress.	(Zhang M. et al., 2023)
	*Medicago sativa L.*	Overexpressing *MsFLS13* promotes flavonoid accumulation, improving salt tolerance.	(Zhang L. et al., 2023)
UV-B stress	*Juniperus rigida*	Low-intensity UV-B enhances phenolic synthesis, while high UV-B hinders it.	(Feng et al., 2025)
	*Rhododendron chrysanthum*	CAD and PAL enzymatic sites were upregulated in response to UV-B stress Lignin accumulation mitigates the harmful effects of UV-B stress. Flavonoids promote plant’s resistance to UV-B stress.	(Gong et al., 2024, 2023; Yu et al., 2024)
	*Zizyphus jujuba*	Ultraviolet radiation improved ROS scavenging ability in Jujube fruits	(Jia et al., 2023)
	*Artemisia argyi*	UV-B stress induces flavonoid biosynthetic genes crucial for stress tolerance	(Gu et al., 2024)
	*Brassica rapa* L. (Pakchoi)	Enhancing flavonoid biosynthetic genes promotes nutritional quality	(Hao et al., 2022; Mao et al., 2024)
	*Morus alba* L.	Flavonoid biosynthesis may contribute to UV-B resistance in Morus leaves.	(Li et al., 2023a)
	*Schisandra chinensis*	Isoquercetin, Quercetin, and 4-hydroxycinnamic acid improved UV-B radiation tolerance.	(Ri et al., 2024)
	*Cajanus cajan* L.	UV-B radiation enhances phenolic deposition	(Gai et al., 2022)
	*Vaccinium corymbosum*	Inhibition of flavonoid accumulation under UV-B stress	(Song et al., 2022)
	*Gossypium hirsutum*	Anthocyanins and lignin were enhanced in response to UV-B stress *Gh*MYB4 negatively regulates anthocyanin to hinder UV-B stress tolerance.	(Song et al., 2025)
	*Salvia verticillata*	Increased expression of *PAL* in young leaves promotes resistance to ultraviolet radiation.	(Rizi et al., 2021)
	*Oryza Sativa*	*OsbZIP18* induces phenylpropanoid biosynthesis for UV-B stress tolerance. *Osbzip18* mutant exhibits reduced phenolic contents under UV-B stress	(Liu X. et al., 2024)
	*Ocimum basilicum* L.	Cold stress enhanced *C4H* expression and other phenolic compounds.	(Rezaie et al., 2020)
	*Brassica rapa* L.	Cold induces polyphenolic compounds involved in ROS-scavenging	(Eom et al., 2022)
Cold stress	*Gastrodia elata*	Increased phenolic activity preserves *G. elata* quality under low-temperature stress.	(Dong et al., 2023)
	*Oryza Sativa*	Negatively regulation of phenylalanine enhanced cold tolerance	(An et al., 2024)
	*Dendrobium officinale*	The upregulation of *F3’H* and *FLS* contributes to cold tolerance in plants	(Zhan et al., 2022)
	*Camellia sinensis*	*CsPAT1* expression increased drought, cold, and heat stress tolerance by regulating phenylpropanoid metabolism (increased flavonoid levels)	(Li J-W. et al., 2025)
Heat stress	*Oryza sativa*	OsUGT72F1-overexpression mediate heat resistance via upregulation of the phenylpropanoid, zeatin, and flavonoid pathway, leading ROS induction	(Ma Y. et al., 2025)
	*Oryza sativa*	*Overexpressing UGT706F1* mediated heat resistance through elevated flavonoid and flavonoid glycosides levels and binds to *MYB61* to form *MYB61-UGT706F1* module increases heat stress resistance	(Zhao et al., 2025)
	*Triticum aestivum*	*TaMGD*-overexpressing wheat plant increased grain weight under elevated heat stress	(Ma D. et al., 2025)
	*Oryza sativa*	*OsPEX1-*overexpressing increases rice vulnerability to heat stress, impairs root growth via increased lignin accumulation and downregulation of Gibberellins	(Li J. et al., 2023; Li J. et al., 2025)
	*Oryza sativa*	Knockout of *OsMAPK3* compromised heat stress resistance	(Deng et al., 2025)

### 3.3 Phenylpropanoids enhance plant responses to temperature stressors

Extremes of temperature retards plant growth and development (Aluko et al., 2021; Ma D. et al., 2025). Plants accumulate more phenolic compounds to detoxify ROS under temperature stress (**Table 3**). Hence, the increased expression of C3H and lignin levels in rhododendron contributes to cold tolerance (Wei et al., 2006). The crucial genes encoding lignin biosynthesis were highly expressed in cold-tolerant cultivars, indicating the contribution of lignin in peach adaptation to cold (Li et al., 2023b). Overexpressing *CaPOA1* and *CaCAD* in *Arabidopsis* increases ROS scavenging and plant tolerance to cold injury (Xiao et al., 2025). A similar increase in phenolic compounds was observed in heat-stressed plants (Commisso et al., 2016; Wang J. et al., 2019; Yuan et al., 2025), indicating the crucial roles of phenylpropanoids in enhancing plants’ tolerance to temperature stress.

### 3.4 Transcriptional regulation of lignin and flavonoids roles in plant defense interactions

While lignin metabolism strengthens the cell wall, enhancing physical resistance to invasion, flavonoid biosynthesis produces essential phytoalexins that support plant immunity and serve as signaling molecules for microbial interactions. For example, the upregulation of the phenylpropanoid pathway under Hrip1 induction conferred resistance to rice blast fungi by reinforcing cell walls through extensive lignin deposition (Ninkuu et al., 2022; Zhang et al., 2021). Wang W. et al. (2025) also reported the enhanced accumulation of lignin against *Tambocerus elongatus* in *Camellia sinensis*. The oxidation of H_2_O_2_ promoted lignin accumulation by downregulating transcriptional inhibitors, including *miR397b*, that adversely regulate *OsLAC7*, *OsLAC28*, and *OsLAC29*, liberating *Copalyl Diphosphate Synthase 2* (*CPS2*) for terpenoids metabolism (Cao and Dong, 2025; Ninkuu et al., 2021). Additionally, a pear plant over-expressing the *PbrMYB14* enhanced lignin accumulation against *Alternaria alternata* and reduced leaf lesions by 68.95% (Yan et al., 2025). *GhBGLU46* has been identified as a key activator of several lignin metabolism genes, including *GhCCoAOMT2*, *GhCCR4*, *GhCAD6*, and *GhCAD.* Thus the overexpressing *GhBGLU46* increased lignin production against Verticillium wilt (Wang et al., 2025a). Li and Wang (2025) also found that *CpVQ20*-overexpressing lines in tobacco promoted flavonoid and lignin metabolism via upregulated *NtF5H* against powdery mildew.

Furthermore, lignin also mediates insect modulation. Recent literature has shown that the overexpressing lines of CCR in Populus enhanced lignin levels to mediate defense against *L. dispar* larvae (Li Y. et al., 2025). *Sl4CLL6* mutant lines hampered the expression of genes downstream of the phenylpropanoid pathway, including *SlHCT*, *SlCAD*, and *SlCHI*, further compromising tomato resistance to mites (Wang et al., 2024).

Flavonoids such as anthocyanins, flavonols, and flavones are highly pigmented and contribute to the flower color of plants (Bisht and Gaikwad, 2025). Recent studies have revealed their novel roles in pest and disease mitigation (Tiwari et al., 2025). Chu et al. (2025) reported the role of *NtWRKY28* in lignin and flavonoid metabolism against aphids in tobacco plants by inducing the upregulation of several phenylpropanoid biosynthetic genes (*PAL*, 4CL, *CHI, CAD*, *HCT*, *CHS*, *C4H*, and *CCR*). Additionally, *VqWRKY56* enhances the transcription of *VqbZIPC22*, which activates salicylic acid and proanthocyanidin metabolism, strengthening resistance to powdery mildew in *Vitis quinquangulari* (Wang Y. et al., 2023). Quercetin accumulation in lima beans also enhances defense against *Tetranychus urticae* (Li F. et al., 2025), while Brown midrib 12 (BMR12) induction promoted COMT activity, increasing JA and flavonoids accumulation against fall armyworm (Kundu et al., 2025). In a study investigating the mechanism of phenylpropanoid’s defense against *Alternaria alternata* in korla fruits, Sun et al. (2025) reported high enzymatic activity of PAL, C4H, and 4CL resulting in significant accumulation of total phenolics, *trans*-cinnamic acid, ferulic acid, caffeic acid, *p*-coumaric acid, and sinapic acid. Notably, higher expression of CHS and CHI significantly improved flavonoid accumulation, including naringenin, rutin, apigenin, quercetin, and epicatechin in defense against *A. alternata* infection. Recent research has highlighted the role of phenylpropanoid metabolism in plant resistance to diseases and pests (**Table 2**).

### 3.5 Phenylpropanoids (Flavonoids) as signaling molecules for root nodulation in legumes

**Figure 4:**
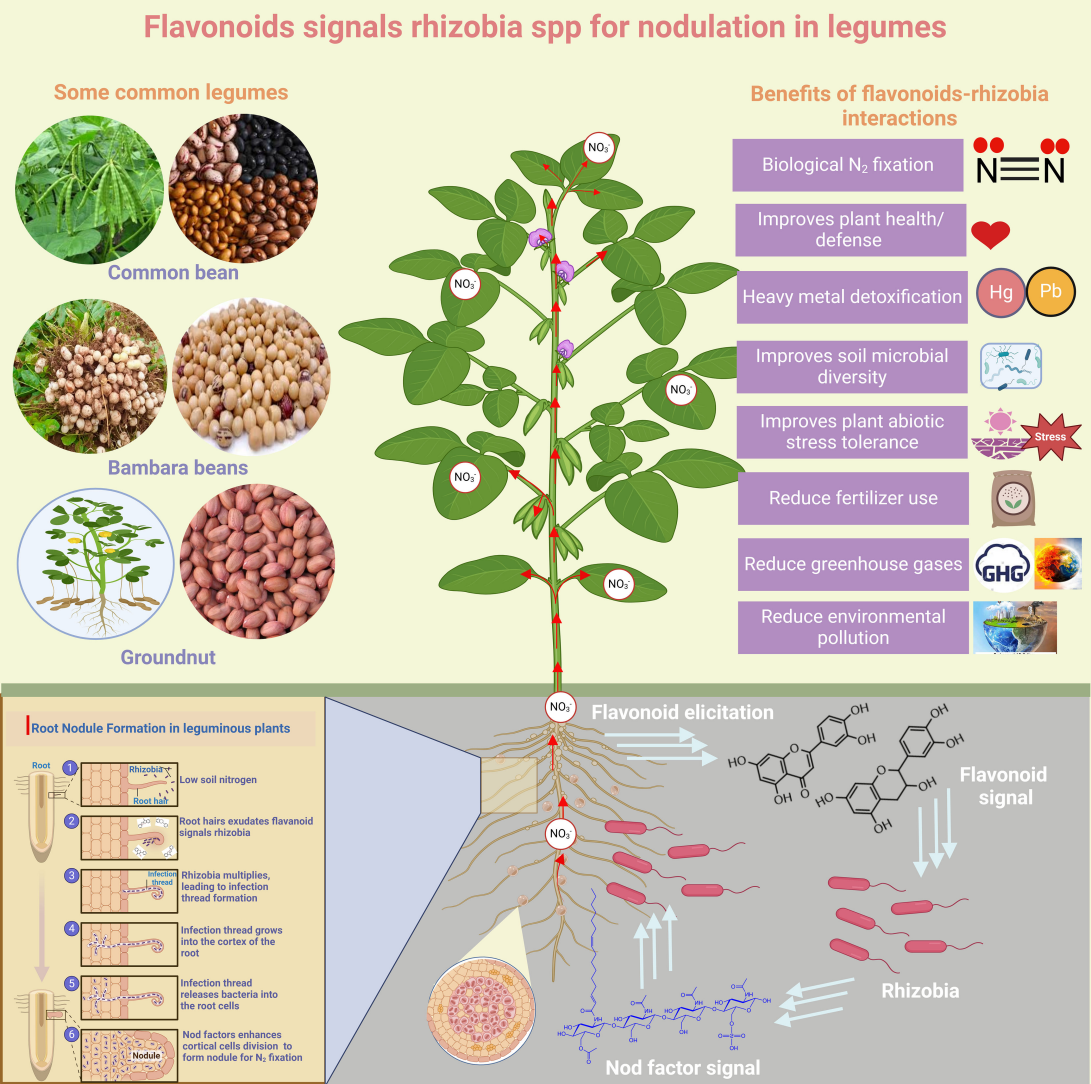
Phenylpropanoids (Flavonoids) signal rhizobia for root nodulation in legumes. The Figure provides an overview of the role of flavonoids as signaling molecules for rhizobia, which infects root hair legumes, leading to nodulation. This symbiotic relationship results in N_2_ fixation for crop growth. The Figure also highlighted the ecological benefits of nitrogen fixation.

Flavonoids play a crucial role as signaling molecules and chemo-attractants in plant-microbe interactions, influencing organisms such as *Fusarium* spp., *Rhizobium*, and arbuscular mycorrhizal fungi. Additionally, they can activate virulence genes in *Pseudomonas syringae* and *Agrobacterium tumefaciens* (Falcone Ferreyra et al., 2012). Flavonoids also play a crucial role in the legume nodulation process (**Figure 3**). Thus, legume roots exudate flavonoids, which rhizobial nodulation (Nod) protein NodD detects, triggering the expression of nod genes and Nod factors (NF) (Ninkuu et al., 2025) (**Figure 4**). NFs induces legume responses for symbiotic interactions (Haskett et al., 2025). Evidence has shown that RNAi of chalcone synthase in legumes exhibited deficiency in nodulation due to the collapse of flavonoid biosynthesis (Abdel-Lateif et al., 2013; Das et al., 2024). Moreover, the *Rlv*3841 NodD regulatory domain deletion line activated NodD_FI_ for transcript accumulation of NF genes (Haskett et al., 2025). Interestingly, flavonoid exudation into the rhizosphere to attract rhizobia spp. is complicated and involves several players. Elicitors have been implicated in inducing flavonoid exudation (Hassan and Mathesius, 2012). However, transgenic Arabidopsis harboring the mutant ABC transporter exhibited altered exudation of flavonoids. ABC transporters have been demonstrated to be involved in isoflavonoid genistein exudation in soybeans, and it has also been reported that flavonoids can be passively released by decomposing roots (Hassan and Mathesius, 2012).

Flavonoids-induced symbiotic interactions between roots of legumes and rhizobia spp. have several ecological benefits (**Figure 4**). Some of these include improvement of soil health, reduction of environmental pollution and GHG emissions from synthetic fertilizer use. Furthermore, ROS accumulation in legume roots upon detecting rhizobia spp. via nod factors can also initiate a crucial signaling cascade (Hérouart et al., 2002). Apart from coordinating symbiotic interaction, ROS production modifies the cell wall and modulates the expression of defense-related genes, positioning legumes’ defense machinery against pathogens. Interestingly, it is currently unknown how cell wall modification favors rhizobia infection but inhibits pathogens.

Two plant growth regulators, cytokinins and auxins, crucially enhance legumes nodulation process, promoting cell division and differentiation (Reid et al., 2017; Ryu et al., 2012). Additionally, cytokinins and auxins promote the growth of root primordia via cell elongation and division in the proliferating zone (Ryu et al., 2012).

## 4 Phenylpropanoids mediate osmotic stress adjustment

Osmotic stresses, such as drought and salinity, are major physiological factors that limit plant growth and yield improvement. The next sections discusses their impacts on plants and highlights the modulatory role of phenylpropanoids in stress response.

### 4.1 Phenylpropanoid biosynthesis is crucial for drought stress tolerance

Drought stress negates various plant physiological processes, ultimately retarding growth and development (Aluko et al., 2021; Jardim-Messeder et al., 2025). Nevertheless, plants have developed adaptative mechanisms for drought, specifically via phenylpropanoid biosynthesis (Rao and Zheng, 2025; Wagay et al., 2023). Earlier studies reported increases in the expression of flavanone-3-hydroxylase (F3H), PAL, 4CL, and flavonol synthase (FLS) enhanced plant tolerance to drought (Chen W. et al., 2025; Ghasemi et al., 2023; Park et al., 2023), perhaps because phenolic compounds mitigates ROS accumulation in the cells, preventing oxidative damage. It has been claimed that flavonoid deposition in the cytoplasm efficiently mitigates the harmful effect of the H_2_O_2_ molecule exerted by drought. However, La et al. (2023) detected a lesser content of flavonoids in soybean under drought stress conditions. Discrepancies in these findings may be influenced by factors including stages of seed development, tissue type, or drought severity (La et al., 2023). Ghasemi et al. (2023) reported a gradual decline in phenolic content, following an initial increase 7 days after drought treatment. Low phenolic formation during the later stages of stress indicates plants’ metabolic adjustment to prolonged stress (Ghasemi et al., 2023). Furthermore, Yan et al. (2023) reported the role of OsOLP1 in mediating rice tolerance to drought via lignin, proline, and abscisic acid accumulation. Elsewhere, Cao P. et al. (2024) identified BGC7 and BGC11 gene clusters consisting of 12 genes, including *4CL*s, *C3H*, *CPA*, and SlMYB13 in phenolamide metabolism against drought stress tolerance in tomatoes, providing deeper insight into crop improvement techniques via genetic engineering and secondary metabolite elicitation. Detailed reports on crop drought-resistant mechanisms mediated by phenylpropanoids metabolism are highlighted in **Table 3**.

### 4.2 Salinity stress tolerance in plants under phenylpropanoid metabolism

Salinity stress is a crucial environmental constraint that halts plant growth and development (Ben Youssef et al., 2025; Safdar et al., 2019; Aluko et al., 2024). High soil salinity decreases leaf dry weight, plant height, photosynthesis, water, and nutrient uptake (Singh et al., 2025; Wang H. et al., 2025). Salt stress promotes the production of ROS, causing oxidative damage to plant cells (Jiang et al., 2025; Singh et al., 2025; Huang et al., 2024; Yang et al., 2023). Therefore, enhancing antioxidant defense systems could contribute to plant salinity tolerance (Ling et al., 2025). One of the most probable ways of improving plants defense system is by increasing the activities of antioxidant enzymes such as CAT and SOD, involved in the removal of H_2_O_2_ and O^2-^, safeguarding against cellular damage (Garcia-Caparros et al., 2021; Shomali et al., 2022). Cao Y. H. et al. (2024) reported a significant increase in SOD and CAT activities under salinity stress, particularly in salt-tolerant genotypes. The salt-tolerant genotype appears to have an in-built phenolic compound, acting as an antioxidant defense system, that scavenges harmful ROS (Cao Y. H. et al., 2024; Bistgani et al., 2019; Chen et al., 2019). Ample evidence revealed that increased expression of phenylpropanoid biosynthetic genes and their respective metabolites contributes to plant salt tolerance (**Table 3**). Increased expression of *NtCHS1* facilitated tobacco tolerance to salt stress (Chen et al., 2019). Flavonoid biosynthetic genes, including *LpFLS1* and *LpCHI1*, highly expressed in ryegrass, suggest their involvement in salt tolerance (Cao Y. H. et al., 2024). Overexpressing *GmCHI4* in soybean enhanced isoflavones content in the salt-stressed root (Zhang J. et al., 2024). These and other findings suggest the contributions of phenylpropanoids in plant salt stress tolerance.

### 4.3 Phenylpropanoids role in postharvest deterioration

Postharvest physiological deterioration (PPD) severely threatens global food security, rendering crops unpalatable 1–3 days after harvest (Chang et al., 2024; Chen Z. et al., 2025; Ji et al., 2025). Different storage methods, including cellular storage, plastic bag wrapping, indoor sand storage, and paraffin wax coatings, have been previously used to improve plants’ postharvest quality. Yet, the interventions are time-consuming and labor-intensive (An et al., 2023; Chang et al., 2024; Chen Z. et al., 2025). Extending the postharvest shelf life is critical for sustainable crop productivity.

Attempts to extend postharvest shelf-life have been quite challenging due to the increased production of reactive oxygen species (ROS), which causes PPD. Phenylpropanoid metabolism has become a crucial defense mechanism to mitigate ROS-induced PPD and improve plant storage stability effectively (Liu Q. et al., 2024; Liu et al., 2017; Wahengbam et al., 2023). Specific phenylpropanoid-derived metabolites, such as phenolics, epicatechin, flavonoids, and ferulic acid, accumulate in stressed or injured plants during storage. Meanwhile, others, including 3,4-flavanone, coumarin, and isoflavone, decrease, suggesting changes in metabolite synthesis contribute to postharvest deterioration under stress. Zheng et al. (2022) recently revealed that changes in the synthesis of phenylpropanoid derivatives impact strawberry postharvest quality under temperature stress. Apple and bulb discoloration have also been attributed to phenylpropane biosynthesis, suggesting phenylpropanoids are crucial for fruit preservation (Chen Z. et al., 2025; Wang J. et al., 2023).

Studies have shown a significant increase in the expression of genes associated with phenolic biosynthesis and ROS turnover during storage, ultimately regulating PPD (An et al., 2023; Liu Q. et al., 2024; Vanderschuren et al., 2014; Wahengbam et al., 2023; Wang B. et al., 2019; Wang C. et al., 2023). Perhaps the reason why PAL expression, which was barely detectable in harvested cassava roots (0hr), increased by 70-fold 72hrs after wounding (Kumar and Knowles, 2003; Wang C. et al., 2023). The enhanced activity of PAL facilitates lignin biosynthesis (Liu et al., 2005); thus, the expression of cinnamate-4-hydroxylase (C4H), which synthesizes precursors of lignin biosynthesis, increased 72hrs after wound healing (Xu J. et al., 2019). Furthermore, 4-coumarate CoA ligase (4CL) facilitates the metabolic flux to flavonoids in PPD-susceptible plants (Wang C. et al., 2023; Wang et al., 2020), indicating the contributions of phenolic compounds in plant storage stability. Recent updates on the crucial roles of phenylpropanoid genes and the respective metabolites are indicated PPD (**Tables 4**, **5**).

**Table 4:** Phenylpropanoid metabolism enhances postharvest deterioration tolerance in crops.

Genes	Plant	Technique	Regulation/ expression	Roles of phenylpropanoids	Reference
*MeC3’H*	Cassava	RNAi	Downregulated	Delayed PPD by decreasing scopoletin and scopoletin accumulation	(Ma et al., 2022)
*StC3’H*	Potato	RNAi	Downregulated	Reduced yield and phenolic metabolites	(Knollenberg et al., 2018)
*MeF6’H*	Cassava	CRISPR-CAS9	Downregulated	Decreased scopoletin levels and PPD symptoms	(Mukami et al., 2024)
*PAL, HCT, CYP98A*, and *PPO1-4*	Lettuce	qRT-PCR	Upregulated	Induced browning in lettuce	(Liu Y. et al., 2022)
*PAL* and *C4H*	Cassava	qRT-PCR	Upregulated	Contributes to wound healing	(Wang C. et al., 2023)
*MeCHS3* and *MeANR*	Cassava	RNAi	Downregulated	Induces cassava tolerance to PPD	(An et al., 2023)
*OsPAL7*, *OsC4H*, and *OsCAD2*	Rice	qRT-PCR	Upregulated	Improves storage stability of paddy rice	(Liu Q. et al., 2024)

**Table 5:** Phenylpropanoid metabolism mediates Postharvest physiological deterioration (PPD).

Metabolite	Plants	Biosynthesis	Function in PPD regulation	Reference
Salicylaldehyde	Cassava	decrease	Low levels of Salicylaldehyde delay PPD	(Drapal et al., 2024)
Chlorogenic acid, Chrysin O-malonylhexoside, Chrysoeriol 7-O rutinoside, calycosin-7-O-glucoside, and glycitin	Lettuce	Increase	Triggers lettuce browning during storage	(Liu Y. et al., 2022; Yang et al., 2022)
Ferulic acid and flavonoids	Bulbs	Increase	maintains the freshness of the bulb	(Chen Z. et al., 2025)
Flavonoids	Cassava	Increase	Induces a delay in cassava PPD	(An et al., 2023)
Anthocyanin	Cassava	Increase	Contributes to PPD resistance	(Drapal et al., 2024)
Flavonoid	Paddy rice	Increase	Improves the storage stability in paddy rice	(Liu Q. et al., 2024)
(-)-Epigallocatechin and L-epicatechin	Cassava	Increase	Induces the severity of cassava PPD	(An et al., 2023)

### 4.4 Interaction between plant growth regulators and phenylpropanoid metabolism

Phytohormones are natural signaling molecules that contribute to plants’ response to environmental cues (Samanta and Roychoudhury, 2025). Recent advances link these naturally synthesized and deployed molecules by plants to the modulatory activity of the phenylpropanoid pathway. For example, ethylene, auxin, strigolactone (SL), jasmonate (JA), and gibberellin are associated with the phenylpropanoid pathway (Silva et al., 2025), indicating the activities influencing phenylpropanoids intricately affect phytohormones. Shi et al. (2024) recent study reported the role of a novel phytohormone, 2,4-dichloroformamide cyclopropane acid (B2) in drought stress tolerance in *Carex breviculmis.* Transcriptome analysis of B2-treated plants activated the expression of drought stress-responsive transcription factors, including AP2/ERF-ERF, WRKY, and mTERF, which consequently upregulated the phenylpropanoid metabolism via the upregulation of *HCT*, *COMT*, and *POD* genes. B2 signaling modulated phytohormone-responsive genes, leading to abscisic acid accumulation for drought tolerance (Shi et al., 2024). Elsewhere, Dey and Sen Raychaudhuri (2024) reported that 1 μM MeJA treatment of *Plantago ovata* enriched the PAL and CHI for enhanced antioxidant defense through ROS signaling, activating significant metabolism of phenolic compounds, such as caffeic acid, chlorogenic acid, vanillic acid, coumaric acid and Luteoloside and PGRs including IAA and GA. Moreover, evidence indicates that *FvTCP9* transcription factor regulates *FaNCED1*, which encodes 9-cis-epoxycarotenoid dioxygenase (NCED), a key enzyme in abscisic acid (ABA) biosynthesis. Activation of *FaNCED1* leads to changes in ABA levels and may be involved in the PYR-PP2C-SnRK2 signaling pathway. Furthermore, *FvTCP9* modulates the transcription of genes associated with anthocyanin biosynthesis (*FaPAL*, *FaCHS*, *FaCHI*, *FaANS*, *FaUFGT*), influencing strawberry coloration. Thus, exploring the intricate interaction of phenylpropanoids and phytohormones can enhance plants developmental cues and stress Reponses.

### 4.5 Phenylpropanoids regulate nutrient deficiency tolerance in plants

Nutrient deficiency stress is one of the leading causes of plant growth retardation and yield loss (Li et al., 2023a, b; Li C. et al., 2023; Ninkuu et al., 2023a). Nitrogen (N), phosphorus (P), and potassium (K^+^) deficiency stress, for instance, disrupt photosynthesis, nutrient uptake, and allocation. Nevertheless, phenylpropanoids mediate plant tolerance to nutrient stress (**Table 6**). Li J. et al. (2023) revealed that the upregulation of flavonoids under N deficiency stress maintains C/N balance of sugarbeet. Low N stress promotes flavonoid biosynthesis, increasing plant enzymatic activities in *snow chrysanthemum* (Li Z. et al., 2023). Similar reports on rapeseed and cassava have shown a significant boost in flavonoid content in response to N deficiency stress (Koeslin-Findeklee et al., 2015; Wang et al., 2025b). Wang et al. (2025b) affirm the upregulation of two CHI in response to low N stress, suggesting CHI is crucial for carbon flux redistribution. Evidence has shown the detrimental impact of N deficiency stress on carbon metabolism, redirecting photosynthetic carbon into the phenylpropanoid biosynthetic pathway (Aluko et al., 2021; Wang et al., 2025b). This shift promotes metabolic flux of the flavonoid downstream genes, resulting in increased flavonoid deposition (Xin et al., 2019). Samarina et al. (2024) reported that increases in lignin and flavonoids improved cell wall stability under N deficiency, suggesting their roles in tea adaptation to low N conditions. Lignin regulates root architecture and other plant physiological processes, and thus, lignin reprograms *Neolamarckia cadamba* root under N deficiency stress (Lu et al., 2021).

**Table 6:** The role of phenylpropanoids in nutrient deficiency mitigation.

Nutrient stress	Plant	Genes/metabolites	Technique	Regulation/expression	Function	Reference
Potassium deficiency	Soybean	Isoflavones and coumestans	UPLC-HRMS	Increased	Isoflavones may be potential biomarkers of K+ deficiency	(dos Santos Cotrim et al., 2023)
Potassium deficiency	*Coconut*	*POD1*, *PER5*, and *PER10*	RNA-seq and qRT-PCR	Upregulated	Lignin biosynthetic genes may participate in low K+ tolerance	(Jin et al., 2024)
Potassium deficiency	Apple	*PAL*, *C4H*, *4CL*, *ANS*, *CHI*, and *CHS*	RNA-seq and qRT-PCR	Upregulated	Flavonoids regulate plant response to low K+ stress	(Sun et al., 2023)
Potassium deficiency	*Brassica napus*	*CAD* and *CCR*	RNA-seq	Upregulated	Phenolics regulate K+ in response to stress	(Liao et al., 2025)
Nitrogen deficiency	*Tobacco*	Lignin biosynthetic genes	RNA-seq	downregulated	Incomplete cell wall development under a low NO_3_^-^ supplyLignin-mediated resistance to aphid infestation.	(Miao et al., 2025)
Nitrogen deficiency	Maize	Cinnamate and flavonoids	LC-MS/MS analysis	Increased	Flavonoids facilitate plant response to Low N stress	(Lu et al., 2023)
Nitrogen deficiency	Cassava	*CHS*, *CHI*, *ANR*, and *F3H*	RNA-seq and qRT-PCR	Upregulated	Flavonoid enhances low N stress tolerance	(Wang et al., 2024)
Nitrogen deficiency	Tea (*Camellia sinensis* L.)	*PAL*, *POD12*, and *CAD3*	qRT-PCR	Upregulated	Phenolics improved cell wall stability under low N stress	(Wang et al., 2024)
Nitrogen deficiency	*Robinia pseudoacacia*	Flavonoids		Increased	Flavonoids contribute to plant adaptation to low N stress	(Li Y. et al., 2024)
Nitrogen deficiency	*Citrus sinensis*	Lignin, flavonoids, phenolic genes	RNA-seq	Upregulated	Phenylpropanoids enhanced N-deficiency tolerance in citrus	(Peng et al., 2023)
Low phosphorus	*Epimedium pubescens*	*FLS*, *C4H*, *4CL*, and *PAL*	qRT-PCR	Upregulated	Flavonoids induce growth in response to low phosphorus stress	(Liu S. et al., 2024)
Low phosphorus	*Neolamarckia cadamba*	Lignin biosynthetic genes, *POD*, and *CAT*	RNA-seq and qRT-PCR	Upregulated	Upregulation of the genes elucidates the response mechanisms to stress	(Zhang et al., 2023a)
Low phosphorus	Peanut	*CAT*, *PAL*, *CCR*, and *POD*	RNA-seq	Upregulated	Lignin biosynthesis maintains plant’s stability under low P stress	(Wu et al., 2022)

Increased activities of phenylpropanoid–derivatives under P deficiency have also been well-documented (Liu S. et al., 2024; Wu et al., 2022). Increased activities of PAL and 4CL suggest their enzymes are crucial downstream metabolites in response to P deficiency stress (Liu S. et al., 2024). Lignin, one of the vital downstream branches, was enhanced in response to P deficiency (Cesarino, 2019). More importantly, lignin biosynthetic genes, including *CCR*, *CAD*, and *POD*, were significantly upregulated in response to low P stress (Wu et al., 2022). Increasing lignin gene expressions may promote cell wall thickening, reduce permeability, and improve plant adaptation to low P stress (Cesarino, 2019).

The impact of phenylpropanoids on low K^+^ stress has been elucidated following the reports of excessive production of harmful ROS upon low K^+^ stress (Sun et al., 2023; Zeng et al., 2015, 2018). Potassium stress increases PAL deposition to detoxify ROS, which damages cell membrane stability (Sun et al., 2023). Moreover, UDP-glucosyl transferase activities have been demonstrated to regulate flavonoid-mediated auxin levels during grain development (Ninkuu et al., 2023b). Although the impact of phenylpropanoids in enhancing plant tolerance to individual stress has been harnessed, less is known under combined N, P, and K stressors (**Table 6**).

## 5 Post‐transcriptional regulation of phenylpropanoid metabolism

Plant cell retains their competitiveness to varying degrees of stress exposure by balancing growth and proliferation expenditures with the stress factors. Under such conditions, plants recruit different levels of gene regulatory activities, such as post-translational and post-transcriptional modification of mRNA, to respond to the stress factors and recovery processes (Hernández-Elvira and Sunnerhagen, 2022). Post-transcriptional gene modification is multi-layered, involving mRNA processing, stability, localization, and protein translations (Courtney, 2021).

The role of Micro RNAs (miRNAs) and small RNAs (RNAs) in targeting the structural genes regulating phenylpropanoid metabolism has been thoroughly studied in relation to plant stress responses (Nayak et al., 2025; Rosatti et al., 2024). MiRNAs modulate their target genes posttranscriptionally through mRNA cleavage or limiting its translation, which is critical in the downstream biochemical pathways and pigment synthesis (Ding et al., 2024). For instance, *miRNA156* modulates flavonoid synthesis by targeting MYB TFs (Rosatti et al., 2024). Additionally, the loss of function of miR-858a liberated the targeting efficiency of flavonoid-specific transcriptional regulators, including *AtMYB12* and *AtCHS1* (Jiang et al., 2021). Moreover, *miR-172*, *miR530*, and *miR157* have been demonstrated to regulate secondary metabolite accumulation in leaves and roots of rice, Arabidopsis, and *Chlorophytum borivilianum* (Jiang et al., 2021). Furthermore, *miR-894*, *miR172*, *miR-9662*, and *miR-166* have also been reported to regulate phenylpropanoid metabolism in plants (Marcela et al., 2019). *MiRNAs*-TFs-target genes complex can upregulate or compromise phenylpropanoid metabolism. SPL9 and SPL13 are targeted explicitly by miR156 to stifle the mRNA level of DFR and inhibit anthocyanin accumulation in the process (Cui et al., 2014; Gou et al., 2011). Nevertheless, *DFR* expression is upregulated for anthocyanin accumulation via overexpressing *miR156*, which inhibits *SPL13* in alfalfa (Feyissa et al., 2019). Our previous study showed that *MiR396b/GRF* module regulates Arabidopsis growth under low sulfur conditions (Ninkuu et al., 2024). Yuan et al. (2024) recent study showed that the *miR396b/GRF6* module improved salt stress tolerance in rice by inhibiting H_2_O_2_ accumulation while elevating ROS-scavenging enzyme activities, including CAT, SOD and POD. Meanwhile, *ZNF9* was identified as a negative regulator of salt stress tolerance by binding to the *miR396b* promoter region. In soybean, *miR398b* targets and represses the transcript level of *GmCCS* and *GmCSD1b*, compromising the defense prowess of the crop. Interestingly, the defense machinery of soybean against *Heterodera glycines* worsened when *miR398b* overexpressing levels were generated. However, silencing of *miR398b* in soybeans improved crop defense capabilities by modulating H_2_O_2_ and O_2_^-^ levels (Zhang X. et al., 2024).

Plant pigmentation can also be regulated by miRNA in plants. Nayak et al. (2025) RNA sequencing identified 74 miRNA regulating white coloration and 61 responsible for brown color pigmentation in cotton by modulating flavonoid biosynthesis.

## 6 Post-translational modification of phenylpropanoid metabolism

Post-translational modifications (PTMs) play a significant role in protein functions, stability, localization, activity, structure, and molecular interactions. Post-translational modifications can also influence lignin biosynthesis and wood formation. Recent studies have demonstrated that PTMs of monolignol enzymes, such as phosphorylation and ubiquitination, inhibit enzymatic activity and stability of proteins (Sulis and Wang, 2020). It is worth noting that PTM of proteins are strongly associated with phenylpropanoid metabolism, including phosphorylation, ubiquitination, glycosylation, and S-nitrosylation. These PTMs are essential for biological processes in plants. For example, Kelch Domain F-Box (KFB) proteins (*KFB1*, *KFB50*, *KFB20*, and *KFB39*) inhibit phenylpropanoid metabolism via PAL ubiquitination and proteasome-mediated degradation. Moreover, *MED5* mediates the activation of *KFB39* and *KFB50*, while KFB^CHS^, which negatively regulates flavonoid biosynthesis, acts as the ubiquitination and degradation of CHS in *A. thaliana* (Kim et al., 2020). Additionally, the ubiquitination of *PAL1–4* reduces KFB proteins, lowering their stability in *Arabidopsis thaliana* via the 26S proteasome. Similarly, the interaction of *OsCCR* with *SCFOsFBK1* in rice decreases its stability through the 26S proteasome (Zhang et al., 2013). Zhang et al. also showed that *MYB156* and *MYB221* interaction with UBC34 diminishes their transactivation of lignin genes and may reduce their stability through the 26S proteasome in *P. tomentosa* (Zheng et al., 2019).

Phosphorylation has long been recognized as a key regulatory modification of proteins. Phosphorylation of *PtrAldOMT2* by SDX deactivates its protein activity in *Populus trichocarpa* by ∼ 60% (Wang et al., 2015). Although R2R3-MYB family members are crucial regulators of gene expression, *PtMYB4* is phosphorylated by *PtMAPK6* during early xylem development (Morse et al., 2009).

## 7 Epigenetic regulation of phenylpropanoid metabolism

Epigenetic regulation, which modifies gene expression without altering DNA sequences, can influence phenylpropanoid metabolism, particularly lignin deposition in plants. Environmental factors can trigger epigenetic modification by altering plant gene expression, leading to phenylpropanoid metabolism as a response factor (Ma H. et al., 2025). Epigenetic regulatory mechanisms preceding lignin and flavonoid metabolism include histone modification, DNA methylation, and miRNA activity (Li W. et al., 2024). For instance, a histone deacetylase *PtrHDA15*, acting as an epigenetic inhibitor, relies on PtrbZIP44-A1 for chromatin histone modifications that repress *PtrCCoAOMT2* and *PtrCCR2* to inhibit lignin accumulation in *P. trichocarpa* (Li W. et al., 2024). Moreover, overexpressing *PtrbZIP44-A1* or *PtrHDA15* triggered the reduction of histone acetylation at *PtrCCoAOMT2* and *PtrCCR2* promoters, leading to reduced lignin accumulation. However, the *ptrbzip44-a1* and *ptrhda15* mutants detected higher histone acetylation levels at *PtrCCoAOMT2* and *PtrCCR2* promoters, triggering the expression of the target gene and lignin deposition (Li W. et al., 2024). The conserved histone H2 variant, H2A.Z, has been shown to negatively regulate anthocyanin biosynthesis in *A. thaliana.* Cai et al. (2019) reported that anthocyanin synthesis in *H2A.Z* deposition-deficient mutants is associated with increased levels of H3K4me3, which is upregulated by anthocyanin-related genes. Furthermore, Peng et al. (2020) demonstrated that virus-induced gene silencing of *McHDA6* (Histone deacetylase 6) inhibited the transcriptional activity of methyltransferase 1 (*McMET1*), leading to enhanced expression of *McMYB10* and increased anthocyanin accumulation in *Malus* crabapple.

## 8 Interaction between phenylpropanoid metabolism and plant signaling pathways

Phytohormones are naturally existing organic signaling molecules that crucially coordinate responses to plant biotic and abiotic interaction and developmental cues at lower concentrations. Plant phytohormones are highly diverse, fulfilling distinct regulatory roles or engaging in complex, multifunctional processes within the plant. They include auxins, cytokinins, Gibberellins (GA), Ascisic Acid (ABA), ethylene, Brassinosteroids, Salicylic Acid (SA), Jasmonates (JAs), and Strigolactones (Chakraborty et al., 2025; Iqbal et al., 2021). It is well-established that phytohormones can regulate phenylpropanoid metabolism, and NAC/MYB has been demonstrated to regulate these hierarchical interactions (Li C. et al., 2024; Li W. et al., 2024). For instance, *PtoJAZ5* is a key regulator of JA-mediated lignin suppression in *Populus*, influencing secondary vascular development. Furthermore, transgenic lines overexpressing *PtoJAZ5* in poplar and *Arabidopsis* exhibited collapsed secondary cell walls attributed to the downregulation of genes involved in SCW formation (Li C. et al., 2024; Li W. et al., 2024; Zhao et al., 2023). Overexpression of *McMYB4* led to increased accumulation of flavonols and lignin in apples. Subsequent Y1H and electrophoretic mobility shift assays (EMSAs) demonstrated that *McMYB4* directly binds to the promoter regions of *McMYB4*, *CAD*, and *F5H*, key genes involved in flavonoid and lignin biosynthesis. Additionally, *McMYB4* was shown to interact with the promoters of AUX/ARF and BRI/BIN genes, thereby activating auxin and brassinosteroid signaling pathways to promote growth and reduce reactive oxygen species (ROS) (Hao et al., 2021). According to Xu C. et al. (2019), overexpression of *PtoARF5.1* and *PtoIAA9m*, which encodes a stabilized form of the IAA9 protein, suppresses secondary xylem development by downregulating genes such as *PAL4* and *WND1B* that are involved in lignin biosynthesis and xylem formation. This repression occurs through inhibiting their positive regulators, *PtoHB7*, *PtoHB8*, and two class III HD-ZIP transcription factors.

Exogenous application of benzylaminopurine and MeJA has also been shown to stimulate the accumulation of proline, ROS, and dehydrins, thereby enhancing antioxidant activity and reinforcing the cell wall with lignin as a physical defense barrier (Avalbaev et al., 2021). Similarly, the application of SA and JA enhanced resistance against drought stress in wheat and French bean via enhanced SOD and POD enzymatic activities, along with the accumulation of defense metabolites, such as anthocyanins, flavonoids, total phenolics, and saponin (Ilyas et al., 2017; Karamian et al., 2020; Mohi-Ud-Din et al., 2021).

## 9 Cutting-edge technologies for optimizing phenylpropanoids commercial production

In recent times, the demand for green bioactive compounds has grown, driven by concerns over the environmental impact of synthetic alternatives. Additionally, the rising global population has stimulated growth in the pharmaceutical and food industries (Adetunde et al., 2025), creating a need for innovative methods to scale up the production of plant-based bioactive compounds. The phenylpropanoid pathway has generated several bioactive ingredients used in fragrance, flavor, food additives, neutraceuticals, and several other drugs (**Table 7**).

**Table 7:** Commercialized phenylpropanoid products, application, and examples of commercial uses.

Compound	Application	Examples of Commercial Use	Citation
Cinnamic acid	Fragrances, cosmetics, flavors, pharmaceuticals	Used in perfumes, synthetic indigo, and anti-inflammatory drugs production	(Vargas-Tah and Gosset, 2015)
*p*-Coumaric acid	UV protection, antioxidants, nutraceuticals	Cosmetics and dietary supplement production	(Yasir et al., 2024)
Ferulic acid	Skincare, food preservation anti-ageing,	Production of photoprotective creams and food antioxidants	(Boo, 2019)
Chlorogenic acid	Antioxidant, anti-diabetic, weight loss products	Coffee-based supplements and cosmetics	(Rodrigues et al., 2023)
Resveratrol	Nutraceutical, cardiovascular health anti-ageing,	Found in supplements and skincare, e.g., skincare serums	(Keylor et al., 2015)
Vanillin	Fragrance, flavoring, pharmaceuticals	Synthetic vanilla flavor	(Fache et al., 2016)
Eugenol	Food additive, Dental care, antiseptic production	Used as an additive on clove oil-based dental anesthetics	(Nejad et al., 2017)
Curcumin	Anti-inflammatory and nutraceutical	Turmeric supplements and other functional foods	(Razavi et al., 2021)
Safrole (shikimol)	flavor and fragrance	Sassafras tea, root beer,	(Lunz and Stappen, 2021)

A range of methods has been used in the commercial production of phenylpropanoids. Traditional approaches, like solvent extraction for vanillin and related compounds, produce only minimal yields. As a result, modern high-yield techniques, such as microbial synthesis, have been developed and adopted. This approach depends on high-titre-tolerant microbes, such as *Escherichia coli* and *Saccharomyces cerevisiae*, as biofactories for the commercial production of phenylpropanoids (Ferulic acid, resveratrol, cinnamic acid) (Vargas-Tah and Gosset, 2015b). For example, heterologous expression PAL/TAL genes in microbes have been used to produce CA and pHCA strains. Under this condition, l-Tyr and l-Phe are transformed into pHCA and CA (Vargas-Tah and Gosset, 2015). Recently, Park et al. (2022) synthesized coniferyl alcohol (CA) and dihydroquercetin (DHQ) by reconstructing the phenylpropanoid pathway in *E. coli*. An *E. coli* strain that produces 187.7 mg/L was engineered to carry phenylpropanoid genes from *A. thaliana*, including *4CL4*, *OMT1*, and *CCR1*. Similarly, naringenin was also produced via 239.4 mg/L of DHQ *E. coli* carrier, harboring *A. thaliana* genes (TT7, F3H, and CPR) (Park et al., 2022).

## 10 Conclusion

Phenylpropanoids are central to plant survival and environmental interactions, serving both structural and chemical roles, as well as biotic and abiotic stress resistance. This pathway has been a crucial target for climate-smart crop development due to the diverse metabolites’ functions in ROS scavenging, UV stress tolerance, salt stress resistance, and extreme temperature tolerance. Based on these functions, plant stress improvement techniques can be carried out to produce crop cultivars that can simultaneously exhibit these traits to enhance food production for the hungry world. Although lignin metabolism in crop plants has generated controversy over the end use of crop straws due to overly recalcitrant to chemical digestion, crop improvement techniques must sustainably engineer lignin pathways to meet crop resilience to stress and industrial application of crop straw. Furthermore, the rapid development of metabolic engineering techniques could benefit the engineering of most of these critical metabolites in the phenylpropanoids pathway for biopesticide development. Conclusively, our review provides a timely update of the current studies on phenylpropanoid metabolism and stress tolerance.
